# Septic Shock Secondary to Tricuspid Valve Vegetation Requiring Surgical Debulking

**DOI:** 10.7759/cureus.45403

**Published:** 2023-09-17

**Authors:** Asher Gorantla, Anandita Kishore, Ugochukwu Ebubechukwu, Meenakshi Narayanaswamy, Harsha Vardhan Taluru, Shruthi Sivakumar, Nimrah Hossain, Suzette Graham-Hill

**Affiliations:** 1 Cardiology, State University of New York Downstate Health Sciences University, Brooklyn, USA; 2 Internal Medicine, Sisters of Charity Hospital, Buffalo, USA; 3 Internal Medicine, State University of New York Downstate Health Sciences University, Brooklyn, USA; 4 Internal Medicine, Veterans Affairs Medical Center, Brooklyn, USA; 5 Internal Medicine, State University of New York Downstate Medical Center, Brooklyn, USA; 6 Neurology, State University of New York Downstate Health Sciences University, New York, USA; 7 Cardiology, Downstate, Brooklyn, USA; 8 Cardiology, Kings County Hospital Center, Brooklyn, USA

**Keywords:** imaging modalities, infective endoc, 2d echo, transesophageal echo, tricuspid valve endocarditis

## Abstract

Tricuspid valve endocarditis is increasing in incidence owing to the prevalent use of intravenous substances. Although most patients respond well to intravenous antibiotics over the course of six weeks, some patients require surgical intervention. A multilayered approach to diagnosis with both transthoracic and transesophageal echocardiography (TEE) is recommended for optimal diagnosis and management. In this article, we report a case of septic shock resulting from tricuspid valve infective endocarditis in a young woman with a history of intravenous drug use who ultimately required cardiothoracic surgical intervention for tricuspid valve vegetation. The sensitivity and specificity of TEE for vegetation on the native valves are about 96% and 90%, respectively. Timely surgical intervention may increase the likelihood of tricuspid valve repair by preventing further destruction of leaflet tissue. Transthoracic echocardiogram (TTE) and TEE have complementary roles in the diagnosis and evaluation of endocarditis. With this case report, we emphasize the importance of multimodality imaging and early surgical intervention to prevent further embolism and destruction of tricuspid valve leaflet tissue.

## Introduction

Tricuspid valve infective endocarditis (TVIE) comprises about 10% of all cases of infective endocarditis. It is usually associated with the use of intracardiac devices, central venous catheters, and intravenous (IV) drug use. Tricuspid valve (TV) endocarditis is increasing in incidence owing to the prevalent use of IV drugs. It is estimated that the incidence of bacterial endocarditis among IV drug users is about 1.5-20 per 1,000 per year [1]. Although most patients respond well to IV antibiotics over the course of six weeks, some patients require surgical intervention. For the detection of TVIE, the sensitivity of TEE for vegetation is about 96% for native valves and the specificity is about 90% [2]. With earlier detection, planning earlier surgical intervention can increase the likelihood of TV repair by preventing further destruction of leaflet tissue. In this case report we emphasize the importance of multimodal imaging techniques in the diagnosis and management of TV endocarditis.

## Case presentation

**Figure 1:**
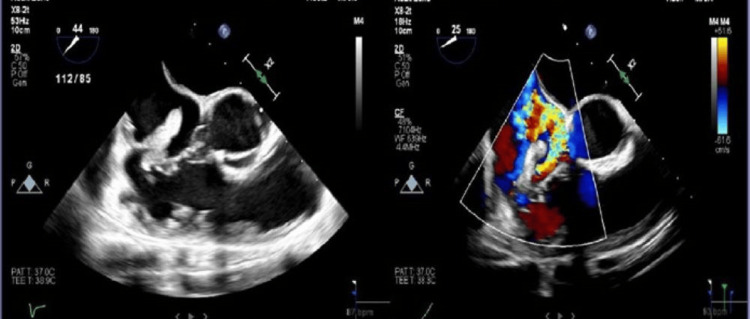
(Left and right) Transesophageal echocardiogram findings of marked thickening of tricuspid leaflets, and large pedunculated, solid, highly mobile vegetation around 2 cm on the body of the septal leaflet with very severe tricuspid regurgitation

**Figure 2:**
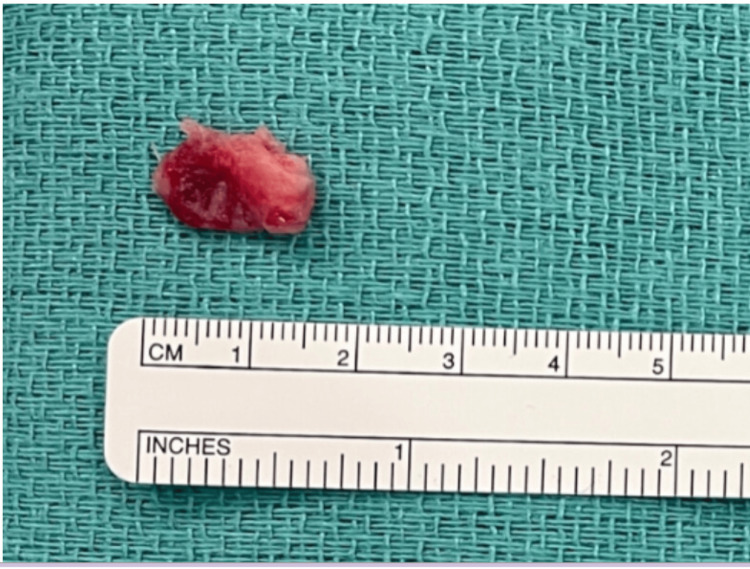
Successful extraction of a 2-cm-sized vegetation from the tricuspid valve using Angiovac device

A 27-year-old woman with a history of IV drug abuse presented with three days of fever and dyspnea. On exam, she was febrile to 103.2F, tachycardic, with mean arterial pressures in the 50s. Her labs were significant for an elevated white blood cell count of 18.86 cells/mm3. After a witnessed generalized tonic-clonic seizure in the emergency department, she was intubated and admitted to the intensive care unit, where she was started on pressors and broad-spectrum antibiotics for septic shock. Computed tomography of the chest showed multifocal pneumonia with nodular opacities consistent with septic emboli. A transthoracic echocardiogram (TEE) showed a large mobile mass on the TV. Blood cultures grew methicillin-sensitive Staphylococcus aureus (MSSA). Her hemodynamic parameters improved, and she was weaned off of pressors and extubated four days later. Transesophageal echocardiogram (TEE) showed marked thickening of the tricuspid leaflets, with severe tricuspid regurgitation and a large, pedunculated, highly mobile 2 cm vegetation on the body of the septal leaflet (Figure 1). Cardiothoracic surgery was consulted and performed a successful Angiovac to remove the vegetation (Figure 2). Repeat TEE showed the absence of previously noted tricuspid vegetation. Post-procedure, she received oxacillin for six weeks.

## Discussion

TVIE accounts for more than 90% of all right-sided infectious endocarditis [2,3]. The incidence of TV endocarditis has been trending upward due to the reported increasing prevalence of IV drug abuse among young adults [4]. IV drug abuse leads to bacteremia via direct inoculation as a result of the breakdown in the body’s innate immune system. Studies have established that patients with IV drug use-related infectious endocarditis are at higher risk of increased rehospitalizations and mortality when compared to patients with non-IV drug use-infectious endocarditis [4,5]. The majority of the identified cases of TVIE have been associated with S. aureus (60%-90%), with other organisms like Pseudomonas aeruginosa, Streptococci, and fungi occurring less frequently [1,2]. S. aureus isolation from blood cultures (as seen in our index patient) carries the highest risk of complications among all possible causative pathogens.

Like all other forms of infective endocarditis, the modified Duke’s criteria have been the widely accepted diagnostic criteria for TVIE. According to the criteria, evidence of two major, one major with three minor criteria, or five minor criteria suggests a definitive diagnosis. Positive blood culture or evidence of endocardial involvement such as vegetation, abscess, or new valvular regurgitation constitute major criteria while the minor criteria include fever more than 38 degrees C (100.4 degrees F), immunologic occurrences such as Roth spots, Osler nodes, glomerulonephritis; predisposing heart defect or IV drug use and vascular phenomena like septic emboli, arterial emboli, conjunctival hemorrhages, splinter hemorrhages, Janeway lesions, etc. [6,7]. Most complications arising from TVIE are due to valvular insufficiency (leading to pulmonary embolism, abscesses, infiltrates, and cavitations as seen in our patient whose chest computed tomography findings were significant for multifocal pneumonia with nodular opacities consistent with septic emboli), valvular stenosis, destruction, and leaflet perforation (leading to septic shock) [1].

Prompt treatment with IV fluids, vasopressors, and empirical IV broad-spectrum antibiotics remains the first line of management in patients with septic shock due to TVIE. Based on the patient’s risk factors, for example, IV drug use, and suspected microorganism(s), empirical IV antibiotics are initiated pending blood culture results and sensitivity. The definitive choice of antibiotics should be tailored towards sensitivity results and usually needed for an extended duration of treatment due to poor penetration of the vegetations/abscesses [6]. Therapy should be followed up with blood cultures every 72 hours to ensure proper eradication of the offending organism(s) [6-8]. Surgical interventions are usually indicated for TVIE patients with microorganisms difficult to eradicate (e.g., persistent fungi), large, persistent TV vegetations, persistent bacteremia for >7 days (e.g., S. aureus, P. aeruginosa) despite adequate antimicrobial therapy, abscess (more common in the setting of prosthetic valve), recurrent pulmonary emboli with or without concomitant right heart failure, and right heart failure secondary to severe tricuspid regurgitation [1,2]. While the American Heart Association/American College of Cardiology (AHA/ACC) recommends early surgery for large mobile vegetation >10mm on a native valve (Class IIb) [7], the European Society of Cardiology (ESC) recommends urgent surgery for isolated vegetation >15 mm and feasible valve repair (Class IIb) [2]. Early surgical intervention is beneficial to the prevention of tissue destruction and further embolization [2,7-10].

## Conclusions

TTE and TEE have complementary roles in the diagnosis and evaluation of endocarditis. Most patients with TVIE are successfully treated with antibiotics. 5%-16% of cases eventually require surgical intervention. The ESC recommends urgent surgery for an isolated vegetation >15 mm and feasible valve repair (Class IIb). The AHA/ACC recommends early surgery for a large mobile vegetation on a native valve (Class IIb). With this case report, we emphasize the importance of multimodality imaging and early surgical intervention to prevent further embolism and destruction of TV leaflet tissue.
